# Expression of NANOG and Its Regulation in Oral Squamous Cell Carcinoma

**DOI:** 10.1155/2020/8573793

**Published:** 2020-07-17

**Authors:** Gašper Grubelnik, Emanuela Boštjančič, Aleš Grošelj, Nina Zidar

**Affiliations:** ^1^Institute of Pathology, Faculty of Medicine, University of Ljubljana, Korytkova 2, 1000 Ljubljana, Slovenia; ^2^Department of Otorhinolaryngology and Cervicofacial Surgery, University Medical Centre Ljubljana, Zaloška 2 and Faculty of Medicine, University of Ljubljana, Korytkova 2, 1000 Ljubljana, Slovenia

## Abstract

**Background:**

Results of previous studies suggest that NANOG may be an important prognostic biomarker in oral squamous cell carcinoma (OSCC), but there are contradictory results regarding NANOG expression patterns on mRNA and protein levels, and the mechanisms of its regulation are poorly understood. Our aim was to analyze the expression and diagnostic significance of NANOG in OSCC, and the possible mechanisms of its regulation, i.e., protein regulators on mRNA level (*OCT4*, *SOX2*, *KLF4*, *AGR2*, and *NOTCH1*), methylation status, copy number variation, and regulatory miRNAs, *miR-145*, *miR-335*, *miR-150*, *miR-34a*, *miR-128*, and *miR-27a*.

**Methods:**

Our study included 120 patients with OSCC. Expression of NANOG protein and mRNA was analyzed using immunohistochemistry and qPCR. Expression of regulatory factors, miRNAs, and copy number variation was performed using qPCR. Methylation status of *NANOG* promoter was determined using PCR and Sanger sequencing.

**Results:**

We detected upregulation of *NANOG* and *OCT4* and downregulation of *NOTCH1* and *AGR2* mRNA in OSCC with lymph node metastases compared to OSCC without lymph node metastases. We observed a strong positive correlation between mRNAs of *NANOG* and those of its protein regulators *OCT4*, *SOX2*, *NOTCH1*, *AGR2*, and *KLF4*. The expression of *NANOG* was in positive correlation with the expression of *miR-34a*. There was also a correlation between T status of OSCC and the expression of *miR-335* and *miR-150* and a correlation of *miR-150* with the N status of T2 OSCC. NANOG promoter methylation and copy number variation were only observed in a small proportion of samples.

**Conclusions:**

Our findings confirm the diagnostic significance of NANOG in OSCC and provide information on NANOG expression patterns on both mRNA and protein levels. They also suggest that protein regulators and microRNAs might play a crucial role, whereas methylation of its promoter and copy number variation probably have a minor role in the regulation of NANOG expression in OSCC.

## 1. Introduction

Despite intensive research, head and neck squamous cell carcinomas still have a very high mortality rate (50%) and an overall 5-year survival of only about 40–50%. The main reasons are late diagnosis, high recurrence rate, and lymph node and distant metastases [1–4]. For Slovenia, the average incidence level for head and neck squamous cell carcinomas from 2011 to 2015 was 463 new cases per year, and oral squamous cell carcinomas (OSCC) represent about half of all head and neck cancer cases in Slovenia [5].

NANOG is an important stem cell transcription factor, with a complex regulation role in human development, determining cell fate, proliferation, and death. After birth, it is expressed at very low levels or is silenced and remains in that state throughout the lifespan. However, NANOG expression is detectable in a proportion of cancer cells that exhibit stem cell-like properties. Cancer stem cells (CSCs) can be the source of malignant transformation, progression, and development of metastases. Elevated NANOG expression has thus been reported in various cancer types (lung, breast, colorectal, etc.), including in OSCC, and it is believed that it can be used as a prognostic biomarker [6–10].

Data available for NANOG expression in head and neck SCC are mostly based on immunohistochemistry or immunofluorescence [11], and they suggest that NANOG expression is upregulated in SCC and associated with tumor development, transformation, and metastasis. Upregulation is correlated with lymph node metastases [2, 4, 12]. It has been suggested that it is important as a prognostic marker [9]. However, little is known about *NANOG* expression on an mRNA level using quantitative real-time PCR (qPCR) [4, 11, 13–16].

Some NANOG regulators have been previously described as being associated with *NANOG* expression in head and neck SCC [2, 12, 17–20]. However, the biological implication and underlying mechanisms still remain unclear and very little is known about the association of microRNAs (miRNAs) and NANOG in OSSC [10, 16, 21–23]. Furthermore, there are limited data on *NANOG* expression in OSCC (i) in correlation with expression of miRNAs whose target is *NANOG*, (ii) in correlation with its known protein regulators, (iii) in correlation with the methylation status of its promoter, and (iv) in correlation with the copy number variation (CNV) of the *NANOG* gene. To understand the regulation of the NANOG in the development and progression of OSCC better, we aimed to characterize the expression of *NANOG* in correlation with its regulators *OCT4*, *SOX2*, *KLF4*, *AGR2*, and *NOTCH1* and with methylation status [24], CNV, and regulatory miRNAs, *miR-145*, *miR-335*, *miR-150*, *miR-34a*, *miR-128*, and *miR-27a* [25–30]. miRNAs were chosen based on miRNA-NANOG interactions in miRTarBase and TarBase v. 8 based on strong evidence validation methods.

## 2. Methods

### 2.1. Patients and Tissue Selection

Our study included 120 patients with OSCC who had been treated surgically, including the resection of the primary tumor and regional lymph nodes, and examination of surgical margins. The study was approved by the National Medical Ethics Committee, Ministry of Health, Republic of Slovenia (No 0120-106/2018/6), and was performed in accordance with the ethical guidelines for human research. The need for informed patient consent was waived, and there is no publication of identifying information or images in the manuscript. Resection specimens were dealt with according to standard procedures and, after 24-hour fixation in formalin, representative samples of the tumor, all lymph nodes, and resection margins were taken, cut at 3-4 *μ*m, and stained with hematoxylin and eosin (HE). p16-positive OSCC presumably related to infection with human papillomavirus infection was not included in the study.

In all cases, corresponding tumor-adjacent normal tissues (TANT) were available. For 90 patients, RNAlater-stored tumor and TANT samples were available, while for 30 patients, only formalin-fixed paraffin-embedded (FFPE) tumor and TANT samples were available. After HE examination, 30 patients with OSCC were divided into 2 groups according to TNM classification.

- Group 1 included 15 patients with early OSCC without lymph node metastases (pT1 or T2 N0 M0); 9 were males and 6 females, aged 32 to 87 years (61.60 ± 14.10). Tumors were located in the tongue (7 patients), floor of the mouth (5 patients), buccal mucosa (2 patients), and alveolar ridge (one patient)
- Group 2 included 15 patients with early OSCC with lymph node metastases (pT1 or T2 N+ M0); 11 were males and 4 females, aged 41 to 76 years (60.53 ± 11.87). Tumors were located in the tongue (9 patients), retromolar trigonum (4 patients), and floor of the mouth (2 patients)

RNAlater (Thermo Fisher Scientific, Waltham, Massachusetts, USA) stored samples were collected constitutively from 2008 to 2018 and divided into 4 groups:

- Group 1 included 29 patients with early OSCC without lymph node metastases (pT1 or T2 N0 M0); 27 were males and 2 females, aged 37 to 84 years (60.14 ± 10.43). Tumors were located in the tongue (23 patients), floor of the mouth (5 patients) and oropharynx (one patient)
- Group 2 included 35 patients with early OSCC with lymph node metastases (pT1 or T2 N+ M0); 30 were males and 5 females, aged 41 to 86 years (57.26 ± 10.25). Tumors were located in the tongue (19 patients), floor of the mouth (7 patients), oropharynx (5 patients), buccal mucosa (two patients), uvula (one patient), and alveolar ridge (one patient)
- Group 3 included 5 patients with advanced OSCC without lymph node metastases (pT3 or T4 N0 M0); all patients were males, aged 42 to 74 years (64.20 ± 12.85). Tumors were located in the tongue (3 patients) and floor of the mouth (2 patients)
- Group 4 included 21 patients with advanced OSCC with lymph node metastases (pT3 or T4 4N+ M0); 20 were males and one female, aged 44 to 77 years (62.43 ± 8.79). Tumors were located in the tongue (11 patients), floor of the mouth (8 patients), oropharynx (one patient), and retromolar trigone (one patient)

### 2.2. Immunohistochemical Detection of NANOG

For immunohistochemistry, 3-4 *μ*m thick slides were cut from FFPE tissue blocks, followed by automated antigen retrieval (BenchMark ULTRA, Ventana, Tuscon, AZ, USA) and staining with commercially available anti-NANOG antibodies (Cell Signaling, cat no. 4903, dilution 1 : 200) (Merck, Kenilworth, New Jersey, USA). Reactions were visualized by incubation with peroxidase and 3,3′-diaminobenzidine (OptiVIEW DAB Detection Kit, Roche, Basel, Switzerland) and then counterstained with hematoxylin. Negative controls omitting the primary antibody binding were included in every run of samples. Testicular seminoma served as a positive control.

We evaluated the extent of positive reaction semiquantitatively (negative-0%, below 25%, between 25 and 50%, between 50 and 75%, and above 75%), intensity (weak, moderate, and strong), and staining pattern (nuclear, cytoplasmic staining, or both).

### 2.3. Isolation of RNA and DNA

#### 2.3.1. Isolation of RNA from FFPE Tissue Samples

For the isolation of total RNA, four 10 *μ*m thick sections were cut and AllPrep DNA/RNA FFPE Kit (Qiagen, Hilden, Germany) was used for total RNA extraction, according to the manufacturer's instructions. All the reagents were from Qiagen, except from ethanol (Merck, Kenilworth, New Jersey, USA) and deparaffinization solution (hexadecane, Sigma-Aldrich, St. Louis, Misuri, USA). NanoDrop-1000 (Thermo Fisher Scientific, Waltham, Massachusetts, USA) was used for measuring the quantity of RNA by measuring A_260_.

#### 2.3.2. RNA and DNA Isolation from RNAlater-Stored Tissue Samples

For each sample, approximately 10 mg of tissue was collected, separately for the isolation of total RNA (including miRNAs), and for DNA. Total RNA extraction was performed using Maxwell® RSC miRNA Tissue Kit (Promega Corporation, Madison, USA), and extraction of DNA was performed using Maxwell® RSC Tissue DNA Kit (Promega Corporation, Madison, USA). Automatic isolation was performed using Maxwell® RSC Instrument (Promega Corporation, Madison, USA), according to the manufacturer's instructions. All the reagents were from Promega except for ethanol (Merck, Kenilworth, New Jersey, USA). Quantity of the RNA and DNA was assessed on NanoDrop-1000 (Thermo Fisher Scientific, Waltham, Massachusetts, USA) by measuring A_260_.

### 2.4. Reverse Transcription (RT)

#### 2.4.1. RT from RNA Extracted from FFPE Tissue Samples

OneTaq-PCR kit (New England Biolabs, Ipswich, Massachusetts, USA) was used to reverse transcribe RNA according to the manufacturer's instructions. Each reverse transcription (RT) reaction contained 60 ng of RNA, 1 *μ*l 6 *μ*M Random primers mix, 5 *μ*l 2x reaction mix and 1 *μ*l 10x enzyme mix. Random primer mix and RNA were mixed and incubated for 5 min at 70°C; then, reaction and enzyme mixes were added and incubated for 5 min at 25°C, 60 min at 42°C, and 4 min at 80°C.

#### 2.4.2. RT from RNA Extracted from RNAlater Stored Samples

RT of total RNA (mRNAs and miRNAs) was performed using miScript II RT (Qiagen, Hilden, Germany), according to the manufacturer's instructions. Each 10 *μ*l RT reaction contained 150 ng of extracted total RNA, 2 *μ*l HiFlex buffer, 1 *μ*l 10x nucleic mix, 1 *μ*l of miScript RT enzyme, and 0.33 *μ*l of RNaze Inhibitor. The reaction was incubated for 60 min at 37°C and 5 min at 95°C.

### 2.5. Preamplification and Quantitative Real-Time PCR

All qPCR reactions for miRNA and mRNA expressions after RT using miScript were performed on ViiA 7 Real-Time PCR System (Applied Biosystems, Foster City, CA, USA). All qPCR reactions for mRNA expression after RT using the OneTaq-PCR kit were performed on Rotor Gene Q (Qiagen, Hilden, Germany). Each sample was done in duplicate.

### 2.6. Preamplification and qPCR of mRNAs

Prior to qPCR, preamplification was performed to measure the expression level of genes, using the TaqMan PreAmp Mastermix (Applied Biosystems, Foster City, California, USA). Preamplification was performed using 2.5 *μ*l of resulting cDNA, 5 *μ*l 2x TaqMan PreAmp Mastermix (Applied Biosystems, Foster City, CA, USA) and 2.5 *μ*l of pooled 0.2x TaqMan Gene Expression Assays. Pooled TaqMan Gene Expression Assays, followed by dilution to 0.2x using Tris-EDTA buffer solution, pH 8.0 (Sigma-Aldrich, St. Louis, Misuri, USA), are summarized in Table 1. Cycling conditions were as follows: 10 min at 95°C and 10 cycles of 15 s at 95°C and 4 min at 60°C.

Efficiency of qPCR reactions was calculated prior to qPCR as follows: (i) pools of N0 and N+ groups for tumors and TANT samples were created by mixing RNA samples isolated from FFPE tissue and (ii) two separate pools of RNA were created, one from healthy mucosa and one from tumor samples, isolated from RNA-stored tissue samples. Resulting preamplified cDNA was diluted 5-, 25-, 125-, and 625–fold for mRNA efficiency analysis using qPCR (described below). All qPCR reactions for efficiency testing were performed in triplicate. For all mRNA expression analyses, TaqMan technology was used.

For FFPE tissue samples, preamplified cDNA was diluted 5-fold. qPCR reaction contained 4.5 *μ*l of diluted preamplified cDNA mixture, 5 *μ*l 2x FastStart Essential DNA Probe Master (Roche, Basel, Switzerland), and 0.5 *μ*l TaqMan Gene Expression Assay (Applied Biosystems, Foster City, CA, USA) (*IPO8*, *SOX2*, *OCT4*, *NANOG*, *NOTCH1*, *AGR2*, and *KLF4*) listed in Table 1. *IPO8* was used as the reference gene. Thermal conditions were applied as follows: 2 min at 50°C, 10 min at 95°C and 40 cycles of 15 s at 95°C, and 1 min at 60°C.

For RNAlater-stored tissue samples, preamplified cDNA was diluted 15-fold. qPCR reaction contained 5 *μ*l of 2x TaqMan Gene Expression Master Mix, 0.5 *μ*l 20x TaqMan Gene Expression Assay (Applied Biosystems, Foster City, CA, USA), and 4.5 *μ*l of diluted preamplified cDNA. Used TaqMan Gene Expression assays are listed in Table 1. *GAPDH*, *IPO8*, and *HPRT1* were used as reference genes. Cycling conditions were as follows: 2 min at 50°C, 10 min at 95°C and 45 cycles of 15 s at 95°C, and 1 min at 60°C.

### 2.7. qPCR of miRNAs

RT and qPCR based on SYBR Green technology were performed to measure the expression level of miRNAs. Prior to qPCR, for the purpose of determination of the efficiency, two separate pools of RNA were created, one from TANT and one from tumor samples. RNA pools were processed in terms of RT and preamplification as described above. Resulting cDNA was diluted 5-, 25-, 100-, 125-, 625-, 1000-, and 3125–fold for miRNA efficiency analysis using qPCR (described below). All qPCR reactions for efficiency testing were performed in triplicate.

For miRNA expression analysis using miScript SYBR Green PCR Kit (Qiagen, Hilden, Germany), cDNA was diluted 150-fold. qPCR reaction contained 5 *μ*l of 2x miScript SYBR Green PCR Mix, 1 *μ*l 10x miScript Universal primer, 1 *μ*l 10x miScript primer, 0.05 *μ*l of ROX dye, 0.95 *μ*l ddH2O, and 2 *μ*l of diluted cDNA. All used 10x miScript primer assays (Qiagen, Hilden, Germany) are listed in Table 2. *SNORA73A*, *SNORD72*, *SNORD61*, and *SNORD95* were used as reference genes. Thermal conditions were as follows: 15 min at 90°C and 45 cycles of 15 s at 94°C, 30 s at 55°C, 30 s at 70°C. Afterwards, melting curves were acquired on the SYBR channel using a ramping rate of 0.7°C/60 s for 60–95°C.

### 2.8. Analysis of Methylation Status of *NANOG* Promoter

Methylation status of the promoter region of *NANOG* was analyzed using Sanger sequencing after bisulfite conversion. Bisulfite conversion was performed using the innuCONVERT Bisulfite Basic Kit (Analytik Jena AG, Jena, Germany) according to the manufacturer's instructions using 500 ng of DNA.

PCR reaction contained 0.2 *μ*l 5 *μ*M primers (forward and reverse) (Sigma-Aldrich, Taufkirchen, Germany), 0.05 *μ*l (5 units/*μ*l) HotStarTaq Plus DNA Polymerase, 1 *μ*l 10x PCR Buffer, 0.7 *μ*l (3.33 mM) dNTPs, 6.35 *μ*l ddH_2_O (Qiagen, Hilden, Germany), and 15 ng bisulfite-converted DNA. Thermal conditions for PCR were as follows: 10 min at 95°C, 40 cycles of 1 min at 95°C, 1 min at 61°C, 1 min at 72°C, and final elongation for 10 min at 72°C. Primers used are listed in Additional file 1: Table S1.

Sanger sequencing was performed on SeqStudio Genetic Analyzer (Applied Biosystems, Foster City, CA, USA). PCR products were directly used for a sequencing reaction after being purified with ExoSAP-IT™ PCR Product Cleanup Reagent (Applied Biosystems, Foster City, CA, USA), according to the manufacturer's instructions. The sequencing reaction was performed using Big-Dye terminator chemistry version 1.1 (Applied Biosystems, Foster City, CA, USA). Electropherograms were analyzed using the Sequence Scanner Software 2, Version 2.0 (Applied Biosystems, Foster City, CA, USA).

### 2.9. Analysis of *NANOG* Copy Number

CNV analysis of *NANOG* was performed using qPCR. Prior to qPCR, for the purpose of determination of the efficiency, two separate pools of DNA were created, one from healthy mucosa and one from tumor samples. As the calibrator sample, the TaqMan Control Genomic DNA (Human male; 4312660; Applied Biosystems, Foster City, CA, USA) was used. Pooled DNA was diluted 2-, 4-, 8-, 16-, and 32–fold starting with 25 ng/*μ*l of DNA. Each qPCR reaction from diluted samples was performed in triplicate (as described below).

For the purpose of *NANOG* CN analysis, multiplex reaction was additionally performed in duplicate for each sample, including *NANOG*-FAM and *TERT*-VIC. Multiplex qPCR was performed using TaqMan Universal Master Mix II, no UGN (Applied Biosystems, Foster City, CA, USA) using 5 *μ*l of 2x TaqMan Universal Master Mix II, no UGN, 0.5 *μ*l 20x *NANOG*-FAM TaqMan CN Assays (Hs04405955_cn), 0.5 *μ*l 20x TaqMan CN Reference Assay (*TERT*-VIC, cat. no. 4403316), 2 *μ*l ddH_2_O, and 2 *μ*l of DNA (6.25 ng/*μ*l). Thermal conditions were as follows: 10 min at 95°C and 40 cycles of 15 s at 95°C, 60 s at 60°C. All reagents were from Applied Biosystems, Foster City, CA, USA. All qPCR reactions were performed on ViiA 7 Real-Time PCR System (Applied Biosystems, Foster City, CA, USA), and each sample was done in duplicate.

### 2.10. Statistical Analysis

For mRNA and miRNA expression analyses, data were analyzed according to Latham [31]. Geometric mean of Cqs of reference genes for mRNAs and for miRNAs was subtracted from mRNAs or miRNAs, to obtain *∆*Cq for statistical analysis.

For FFPE tissue samples, for which TANT was available, statistically significant difference was calculated using the *∆*Cq and Wilcoxon signed rank tests. Further, *∆*Cq of the tumor sample was first subtracted from *∆*Cq of corresponding TANT to calculate *∆∆*Cq (*∆∆*Cq = *∆*TANT − *∆*Cq tumor tissue). *∆∆*Cq of OSCC N0 and OSCC N+ was compared using the Mann–Whitney test. *ΔΔ*Cq was used for calculating fold change using 2^-*ΔΔ*Cq^ for graphical presentation.

For RNAlater-stored tissue samples, comparison of relative quantification of mRNAs and miRNAs (*Δ*Cq) [32] between independent groups of samples was performed using the Mann–Whitney *U* test. For comparison of methylation status of the promoter region of *NANOG* and CNV of *NANOG* to *NANOG* expression, T status and N status, the Spearman rank-order correlation was performed. The Spearman rank-order correlation was also used for all other nonparametric correlations/associations.

Statistical analysis of data was performed using IBM SPSS Statistics 24 software (SPSS Inc., Chicago, IL, USA). Differences were considered as significant using cut-off *p* < 0.05 (2-tailed).

## 3. Results

### 3.1. Expression of *NANOG*, *OCT4*, *SOX2*, *AGR2*, *KLF2*, and *NOTCH1* in OSCC with and without Lymph Node Metastases (FFPE Tissue Samples)

Although a previous study [33] on a cohort of 30 selected FFPE tissue samples was successful in terms of analyzing miRNA expression, the quality of isolated RNA was poor for mRNA expression analyses. We were therefore able to analyze the expression of *NANOG*, *SOX2*, *OCT4*, *AGR2*, *KLF4*, and *NOTCH1* in only 11 tumor samples and corresponding TANT (22 FFPE tissue samples).

Paired FFPE tissue samples (tumor sample and corresponding TANT) were compared using the *∆*Cq and Wilcoxon signed rank tests. Expression of genes in OSCC N0 compared to TANT showed downregulation of *NANOG*, *OCT4*, and *KLF4* (5.86-fold, *p* = 0.018; 3.30-fold, *p* = 0.018; 1.24-fold, not significant, respectively) and upregulation of *SOX2*, *AGR2*, and *NOTCH1* (1.94-fold, not significant; 15.78-fold, not significant; 2.48-fold, *p* = 0.028, respectively). On the other hand, in OSCC N+, expression of *NANOG*, *OCT4*, and *AGR2* showed upregulation (18.42-fold, 6.65-fold, and 4.46-fold, respectively) and *SOX2*, *KLF4*, and *NOTCH1* showed downregulation (1.53-fold, 1.02-fold, and 3.0-fold, respectively), not significant. The *p* value of the difference of *NANOG*, *OCT4*, and *NOTCH1* expression levels between OSCC N+ and corresponding TANT groups was just above the cut-off (*p* = 0.068).

**Figure 1:**
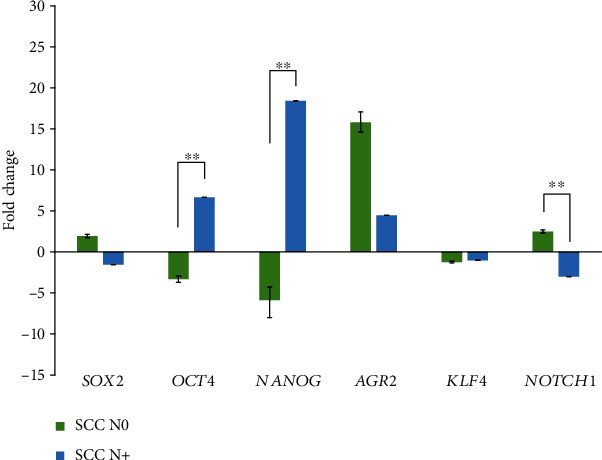
Fold change values for all tested genes in formalin-fixed paraffin-embedded samples. Abbreviations: N0: lymph node metastases absent; N+: lymph node metastases present; SCC: squamous cell carcinoma; ^∗∗^correlation is significant at the *p* ≤ 0.01 (2-tailed).

Using the Mann–Whitney test, comparison of *∆∆*Cq of OSCC N0 (*n* = 7) and OSCC N+ (*n* = 4) revealed that expression of *NANOG* and *OCT4* was significantly upregulated in OSCC N+ compared to OSCC N0 (*p* = 0.008). In contrast, *NOTCH1* was significantly downregulated (*p* = 0.011). For *SOX2*, *AGR2*, and *KLF4*, there were no statistically significant differences, although *AGR2* and *SOX2* showed similar expression pattern. Results are presented as fold change in Figure 1.

Expression of *NANOG* was in positive correlation with the expression of *OCT4* in TANT and tumor samples (*r*_s_ = 0.846, *p* = 0.001; *r*_s_ = 0.713, *p* = 0.009, respectively).

### 3.2. Immunohistochemical Expression of NANOG in Tumors

**Figure 2:**
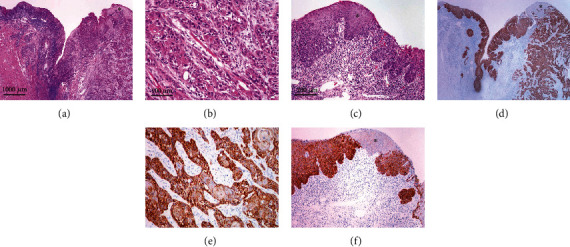
(a) Squamous cell carcinoma (right) and adjacent mucosa with severe dysplasia (left) and a small area of mild dysplasia (^∗^). HE, orig. magnification 2x. (b) Invasive squamous carcinoma. HE, orig. magnification 20x. (c) Adjacent mucosa with severe dysplasia and a small area of mild dysplasia (^∗^). HE, orig. magnification 10x. (d) Expression of NANOG in carcinoma and in dysplastic epithelium in adjacent mucosa (^∗^). Immunohistochemistry, orig. magnification 2x. (e) Expression of NANOG in carcinoma, with cytoplasmic staining. Immunohistochemistry, orig. magnification, 20x. (f) Strong staining for NANOG in areas of severe dysplasia and weak staining in areas of mild dysplasia (^∗^). Immunohistochemistry, orig. magnification 10x.

Thirty cases of OSCC were stained immunohistochemically for NANOG; a positive reaction was found in 27 cases. Immunohistochemical staining was present in the cytoplasm of tumor cells; no nuclear staining was observed (Figure 2). The extent of positive reaction varied, being present in more than 75% of the tumor in 22 cases (81.5%), between 50 and 75% in 3 cases (11.1%), and between 25-50% in 2 cases (7.4%). The intensity of staining was weak in 7 cases (26.0%), moderate in 8 cases (29.6%), and strong in 12 cases (44.4%). In adjacent mucosa, positive staining was found in areas of dysplastic epithelium, staining was intense in severe dysplasia, and staining was very weak in mild dysplasia (Figure 2). Occasionally, staining was more intense in dysplastic mucosa adjacent to OSCC than in OSCC itself. No staining was found in morphologically normal mucosa (Supplementary file 1: Figure S1). In testicular seminoma, which was used as positive control, only nuclear staining was present in all tumor cells.

### 3.3. Expression of *NANOG* in Tumors and Corresponding Normal Tissue

*NANOG* mRNA was present in all tumor samples tested; however, it was undetectable in 10 TANT samples. We observed a stable expression of reference genes in 82 of 90 RNA samples isolated from RNAlater-stored tissue samples of OSCC. These 82 samples were used for further analysis to obtain reliable results.

Due to extremely high variability in Cq values among mRNAs, miRNAs, and reference genes isolated from TANT in comparison to tumor samples stored in RNAlater, TANT samples were not appropriate for further analysis.

Another technically challenging question in the case of TANT was the expression of *NANOG*. It is expected that mRNA of *NANOG* would not be detected in TANT, since there is no protein expression. However, we observed *NANOG* expression in TANT using qPCR, but the technical variability was similar to that of reference genes.

Due to both of the above-mentioned reasons, we excluded samples of normal mucosa from further statistical analyses and comparisons.

### 3.4. Expression of *NANOG* and *SOX2*, *OCT4*, *KLF4*, *AGR2*, and *NOTCH1*

Using the Spearman rank-order correlation (2-tailed), strong positive correlation between *NANOG* and all the tested regulators was observed in tumor samples as follows: *OCT4* (*r*_s_ = 0.395, *p* = 0.001), *KLF4* (*r*_s_ = 0.570, *p* = 0.001), *NOTCH1* (*r*_s_ = 0.515, *p* = 0.001), *SOX2* (*r*_s_ = 0.292, *p* = 0.008), and *AGR2* (*r*_s_ = 0.219, *p* = 0.048).

**Figure 3:**
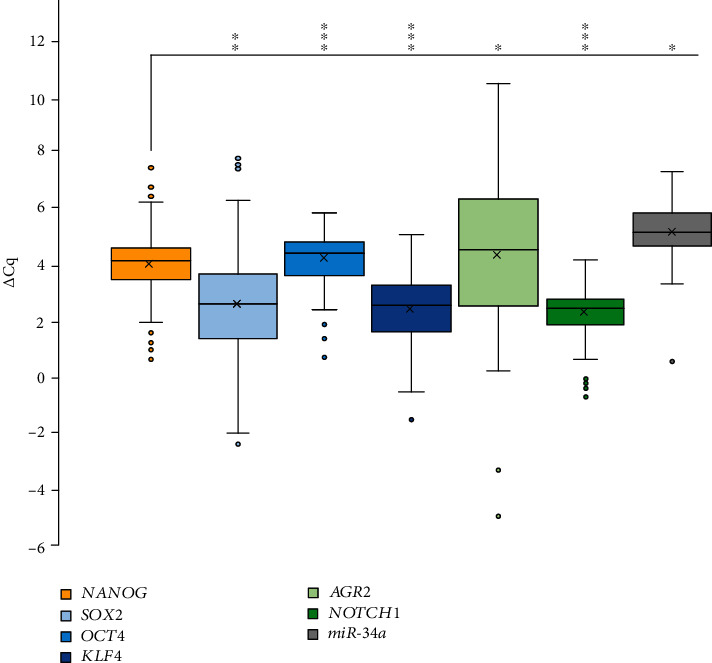
Correlations of NANOG and its regulators. Abbreviations: *∆*Cq: delta quantitation cycle; ^∗^correlation is significant at *p* ≤ 0.05 (2-tailed); ^∗∗^correlation is significant at *p* ≤ 0.01 (2-tailed); ^∗∗∗^correlation is significant at *p* ≤ 0.001 (2-tailed).

We observed correlation also among expressions of other mRNAs beside *NANOG*. Positive correlation was observed between expressions of *SOX2* and *AGR2* (*r*_s_ = 0.486, *p* = 0.001) and between expressions of *SOX2* and *KLF4* (*r*_s_ = 0.322, *p* = 0.003). Expression of *OCT4* was positively correlated with expression of *KLF4* (*r*_s_ = 0.318, *p* = 0.001) and *NOTCH1* (*r*_s_ = 0.442, *p* = 0.001). Expression of *KLF4* was in positive correlation with expression of *NOTCH1* (*r*_s_ = 0.482, *p* = 0.001) and expression of *AGR2* (*r*_s_ = 0.269, *p* = 0.02). All correlations are summarized in Figure 3 and Supplementary file 1: Table S2.

### 3.5. Expression of *NANOG* and *miR-145*, *miR-335*, *miR-150*, *miR-34a*, *miR-128*, and *miR-27a*

We observed statistically significant correlation between expression of *NANOG* and *miR-34a* (*r*_s_ = 0.261, *p* = 0.018) (Figure 3 and Supplementary file 1: Table S2). On the other hand, there were no correlations between expression of *NANOG* and other miRNAs (*miR-145*, *miR-335*, *miR-150*, *miR-128*, and *miR-27a*) in tumor samples.

However, we observed correlation between expressions of investigated miRNAs with other genes that have been recognized as regulators of *NANOG* expression. Expression of *OCT4* was correlated significantly with expression of *miR-34a* (*r*_s_ = 0.247, *p* = 0.03) and *miR-27a* (*r*_s_ = 0.265, *p* = 0.02). Expression of *NOTCH1* was correlated with expression of all investigated miRNAs, except *miR-128*, with *miR-150* (*r*_s_ = 0.428, *p* = 0.001), *miR-34a* (*r*_s_ = 0.290, *p* = 0.01), *miR-145* (*r*_s_ = 0.233, *p* = 0.04), *miR-335* (*r*_s_ = 0.254, *p* = 0.02), and *miR-27a* (*r*_s_ = 0.250, *p* = 0.02).

**Figure 4:**
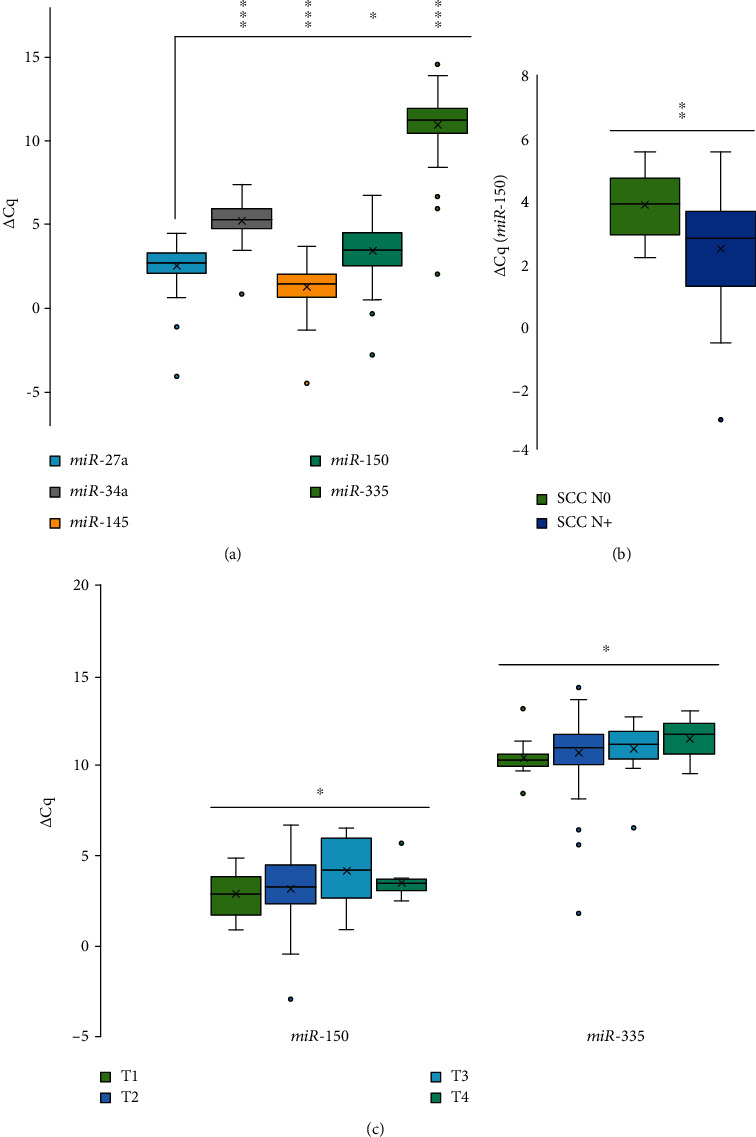
Expression of miRNAs in tumor samples. (a) Correlations between tested miRNAs in tumor samples; (b) difference in expression of miR-150 in T2 tumor stage samples between tumors with and without lymph node metastases (*n* = 45); (c) correlation between the T status of tumor samples and expression of miR-335 and miR-150. Abbreviations: *∆*Cq: delta quantitation cycle; ^∗^correlation is significant at *p* ≤ 0.05 (2-tailed); ^∗∗^correlation is significant at the *p* ≤ 0.01 (2-tailed); ^∗∗∗^correlation is significant at p ≤ 0.001 (2-tailed). N0: lymph node metastases absent; N+: lymph node metastases present; SCC: squamous cell carcinoma.

Expression of all the tested miRNAs was in correlation to each other in tumor samples. Expression of *miR-145* was correlated with expression of *miR-335* (*r*_s_ = 0.691, *p* = 0.001), *miR-34a* (*r*_s_ = 0.796, *p* = 0.001), *miR-128* (*r*_s_ = 0.592, *p* = 0.001), *miR-27a* (*r*_s_ = 0.646, *p* = 0.001), and *miR-150* (*r*_s_ = 0.253, *p* = 0.022). Expression of *miR-335* was correlated significantly with expression of *miR-150* (*r*_s_ = 0.415, *p* = 0.001), *miR-34a* (*r*_s_ = 0.641, *p* = 0.001), *miR-128* (*r*_s_ = 0.552, *p* = 0.001), and *miR-27a* (*r*_s_ = 0.548, *p* = 0.001). Expression of m*iR-150* was significantly correlated with expression of *miR-34a* (*r*_s_ = 0.376, *p* = 0.001), *miR-128* (*r*_s_ = 0.337, *p* = 0.01), and *miR-27a* (*r*_s_ = 0.265, *p* = 0.02). There was also correlation between expression of *miR-34a* and expression of *miR-128* (*r*_s_ = 0.606, *p* = 0.001) and *miR-27a* (*r*_s_ = 0.765, *p* = 0.001). Expression of *miR-128* was also correlated with expression of *miR-27a* (*r*_s_ = 0.567, *p* = 0.001). Some results are summarized in Figure 4(a).

### 3.6. Correlation of mRNAs and miRNA Expression with pTNM Stage

There was a correlation between the T status of tumor samples and expression of *miR-335* (*r*_s_ = 0.236, *p* = 0.03) and *miR-150* (*r*_s_ = 0.219, *p* = 0.05) (Figure 4(c)). No correlation was observed between N status of tumor samples and miRNAs. There was also no correlation observed between expression of mRNAs and T or N status.

When considering only tumor stage T2 (*n* = 45), statistically significant change in expression was observed for *miR-150* between OSCC with and without lymph node metastases (*p* = 0.006) (Figure 4(b)).

### 3.7. Methylation Profile of NANOG Promoter Region

Promoter region of NANOG-201 transcript (ENST00000229307.9; >12 dna:chromosome chromosome:GRCh38:12:7787400:7791801:1) was used as reference sequence.

We analyzed methylation profile of 41 CpGs in *NANOG* promoter region of 10 tumors and TANT samples (1 PT1 N0, 1 PT1 N+, 3 PT2 N0, 2 PT2 N+, 1 PT3 N0, 1 PT3 N+, and 1 PT4 N+). We noticed two additional CpG sites in our samples, numbered by 22 (A->G) and 29 (T->G) (Supplementary file 1: Figure S2). We compared sequences of all the CpGs for each pair of tumor and TANT, and then, we calculated the ratio of samples with differences in methylation status for each CpG (Supplementary file 1: Figure S3). However, there are sites with no differences between tumors and TANT (sites 23-28, 34-36).

We also calculated the ratio of sites with differences in methylation between tumors and TANT for each sample separately. Then, we calculated the correlation between the methylation ratio of NANOG promoter and expression of *NANOG* in tumor samples using the Spearman rank-order correlation. There was no statistically significant correlation.

### 3.8. *NANOG* Copy Number Variation

CN of *NANOG* was analyzed in 87 samples; results are presented in clustered columns for each tumor and TANT, arranged from T1–T4 tumor status and additionally in each group from N0 to N+ status (Supplementary file 1: Figure S4). There is a difference in *NANOG* CN comparing tumor and TANT. We calculated the share of samples with greater CN in tumor samples (*n* = 29; 33.33%) compared to samples where CN was greater in TANT compared to tumor samples (*n* = 58; 66.67%). We also calculated average CN for tumor samples (3.45 ± 0.96) and for TANT (3.63 ± 0.47), regardless of N status. There is a difference in CN greater than one copy comparing tumor samples and TANT in 17 samples (19.54%); in contrast, there is a difference in CN lower than one copy in 70 pairs (80.46%).

No correlation was observed between expression of *NANOG* and *NANOG* CNV of tumor samples using the Spearman rank-order correlation.

## 4. Discussion

We analyzed the expression and regulation of *NANOG* in OSCC and confirmed its important role in the development and progression of OSCC. As expected, NANOG was not expressed in normal tissue but it was expressed in OSCC, as already reported in many types of human cancers, including head and neck cancer [34]. Furthermore, *NANOG* expression was significantly higher in OSCC patients with nodal metastases than in those without nodal metastases, suggesting its role as a prognostic biomarker. Immunohistochemical analysis of NANOG expression showed positive staining in a large proportion of OSCC cases, with a cytoplasmic pattern, in contrast to the nuclear pattern observed in germ cell tumors. The significance of the two different staining patterns remains unclear. The three negative cases, interestingly, were more than 10 years old, which might had influenced the immunohistochemical staining. In areas of the dysplastic epithelium adjacent to OSCC, positive staining was found, being very weak in mild dysplasia and intense in severe dysplasia. Occasionally, staining was more intense in the dysplastic mucosa adjacent to OSCC than in OSCC itself.

Despite an important role of NANOG in the development and progression of OSCC, little is known about the regulation of its expression. We therefore studied possible mechanisms and factors of NANOG expression regulation, such as protein regulators on the mRNA level, microRNAs, CNV, and methylation status.

On the basis of previous studies of NANOG regulators in various cancers, we chose the following protein regulators for further analysis: AGR2, KLF4, NOTCH1, OCT4, and SOX2. We found a strong positive correlation between *NANOG* and mRNA levels of all the tested protein regulators in tumor tissue samples, indicating that these regulators affect *NANOG* expression in OSCC, too. We preliminarily unraveled the correlation in the expression between *NANOG* and other stemness factors.

Previous studies were mainly focused on the expression of two transcription factors, OCT4 (*POU5F1*) and SOX2, which regulate *NANOG* expression through binding to its promoter. *NANOG*, *OCT4*, and *SOX2* mRNA expressions have previously been shown to be significantly higher in samples of moderately and poorly differentiated compared to well-differentiated squamous cell carcinoma [13–15]. Our results are consistent with previous studies that have shown that *OCT4* and *SOX2* are correlated with *NANOG* in OSCC. Another analyzed transcription factor, *KLF4*, has been reported to be associated with both oncogenesis and tumor suppression [2, 13, 19]. *OCT4* and *SOX2* in cooperation with *KLF4* can activate *NANOG* promoter synergistically [18]. It has been suggested that *KLF4* expression can be used as a poor prognostic factor in head and neck cancer [13].

NOTCH1 is a transmembrane isoform receptor, associated with poor prognosis. Similar to our data on *NOTCH1* mRNA expression, immunohistochemical analysis of NOTCH1 protein expression revealed its higher expression in OSCC samples. NOTCH1 is an important factor involved in epithelial-mesenchymal transition, which is believed to be an initial step in the process of forming cancer metastasis, as well as a mechanism responsible for stem cell properties [12].

Another NANOG regulator, AGR2, is involved in tumorigenesis and progression of multiple human cancers. Using head and neck SCC cell lines and *AGR2* knockdown, *NANOG* and *OCT4* downregulation was observed. mRNA expression of *AGR2* was shown to be significantly increased in head and neck SCC in comparison to normal mucosa. AGR2 protein expression correlated with high grade head and neck SCC, with T status and with lymph node metastasis [20]. To the best of our knowledge, our study is the first to show a positive correlation between *AGR2* and *NANOG* on the mRNA level.

Little is known about other mechanisms of NANOG regulation. We therefore analyzed *NANOG* expression in correlation with the methylation status of promoter, CNV, and the expression of regulatory miRNAs, in order to understand the regulation and role of NANOG in the development of OSCC and to evaluate the importance of NANOG as a diagnostic marker [2, 4, 11–21, 23–28].

Candidate miRNAs for our research were selected using TarBase v. 8 and miRTarBase, based on validated miRNA-*NANOG* interactions [35, 36]. Selected miRNAs including *miR-27a*, *miR-34a*, *miR-128*, *miR-145*, *miR-150*, and *miR-335* may act as tumor suppressors, because they have already been associated with direct or indirect regulation of *NANOG* expression and epithelial-mesenchymal transition regulation in various cancers [8, 26, 29, 30, 34, 37–44]. All analyzed miRNAs were expressed in OSCC and TANT (data not shown). However, only *miR-34a* correlated positively with *NANOG* expression in our OSCC samples. Downregulation of *miR-34a* and upregulation of *NANOG* expression in OSCC have been previously reported, but with conflicting results [43–45]. Another fact is that *miR-34a* is a *p53*-responsive miRNA, so it is not surprising that we also found correlation of its expression with *OCT4* and *NOTCH1*.

Other microRNAs also showed promising results: we observed a correlation between the expression of *miR-150*, *miR-145*, *miR-335*, and *miR-27a* and mRNAs of *OCT4* and *NOTCH1* but not with *NANOG*. Interestingly, there is no evidence in the literature that these miRNAs might regulate or be regulated by *OCT4* or *NOTCH1*, but some of them have been reported to play a role in various cancers. For example, *miR-27a* has been reported to promote proliferation, invasion, and migration in colorectal cancer. Furthermore, *miR-27a* has been suggested to be involved in the regulation of *SOX2* in OSCC and, consequently, indirectly to inhibit epithelial-mesenchymal transition. However, the role of *miR-27a* in OSCC remains largely unknown [37]. *miR-145* has been suggested to act as a tumor suppressor in human cancers. In some cancers, *miR-150* acts as an oncogene and is significantly upregulated and has been positively correlated with metastasis and tumor recurrence. Esophageal SCC has shown association of low *miR-150* expression with lymph node metastasis, lymphatic invasion, and poor prognosis [40]. We also observed a correlation between *miR-150* expression and tumor size, as well as lymph node metastases in OSCC. *miR-335* that was correlated to the tumor size of OSCC has been identified as a metastasis suppressor miRNA [26].

Transcription can be regulated by cytosine methylation within promoter regions, which is an epigenetic modification with an important role in gene silencing. This promoter region contains five CpG-dinucleotides, previously reported to be important for silencing *NANOG* promoter by methylation and correlated to the cell differentiation state [24]. Our results suggest that these same five CpGs are also important in tumor progression in OSCC, since we observed a 30-80% difference in methylation status when comparing tumor and TANT. Additionally, we observed a difference in methylation status in other CpG sites and some CpG sites with no difference. However, there was no significant correlation between methylation status and mRNA expression of *NANOG*. We suggest that methylation of *NANOG* promoter is involved in its regulation during OSCC development, at least to certain degree, and is part of the complex regulation of *NANOG*.

The *NANOG* gene is located on the 12 chromosome, at 12p13.31. This region is often exposed to duplications and amplifications in human tumors, resulting in two copies of *NANOG* present in the genome; one is *NANOG* and the other the pseudogene *NANOG2* (*NANOGP1*). Copies can be identical, occurring in 97% of cases, but their transcripts are often differentially spliced [6, 8, 46]. Analyzing CNV of *NANOG* in OSCC samples, there were on average four copies of *NANOG* in most of the tested samples. We found that there is a difference in *NANOG* CNV in tumor samples compared to TANT; however, it seems that *NANOG* CNV has a certain role in its regulation but is not crucial for tumorigenesis.

In addition to NANOG, which was the main focus in this study, there are also other important known stemness regulators in head and neck SCC including OSCC, such as stem cell markers (e.g., CD24, CD44, ALDH1, and STAT3). Among known stemness transcription regulators, transcription factors that are inducers of epithelial-mesenchymal transition (e.g., SNAIL, SLUG, and TWIST) might also lead tumor cells to achieve stem cell-like properties [2, 33, 47]. However, their expression was beyond the scope of the current research.

The present study has several strengths and limitations. The main strength is related to our approach, which enabled insight into NANOG expression on both mRNA and protein levels and various possible mechanisms and factors of its regulation. Another strength is related to the fact that our study included both FFPE tissue and tissue stored in RNAlater. In FFPE tissue, nucleic acids are fragmented and therefore difficult to analyze, but a great advantage of FFPE tissue is that all samples are first evaluated by pathologists, enabling appropriate diagnosis. In our study, only samples that had successfully passed initial quality control with stable expression of reference genes were further analyzed, thus limiting the number of included samples. In contrast, another limitation was in tissue stored in RNAlater, where nucleic acids are well-preserved, but pathohistological analysis is not possible and samples taken from macroscopically looking normal mucosa may contain foci of dysplasia or even carcinoma, as shown in Figure 2. This is probably why analysis of *NANOG* in TANT showed highly variable values, from undetectable expression to high expression similar to invasive carcinoma. We therefore excluded samples of normal mucosa from further statistical analyses and comparisons. Another limitation was the subjective cut-off when stratifying our samples and also for immunescoring results. We were only able to analyze NANOG in relation to TNM status of our samples as we described. However, in view of the new General Data Protection Regulation, we were unable to obtain additional patient information for prognostic value evaluation. We focused on mRNA expression levels of NANOG protein regulators and that is a limitation, since mRNA correlation does not necessarily represent protein correlation.

## 5. Conclusions

Our findings confirm the diagnostic significance of NANOG in OSCC and provide information on NANOG expression patterns on both mRNA and protein levels. They also suggest that NANOG protein regulators and miRNAs play a crucial role, whereas methylation of its promoter and CNV probably have a minor role in its regulation. Further studies should focus on the diagnostic and prognostic significance of NANOG and its regulators in the whole process of cancerogenesis, including preneoplastic stages.

## Figures and Tables

**Table 1:** TaqMan gene expression assays.

Gene symbol	Assay ID	Catalogue no.
*GAPDH*	Human GAPDH	4310884E
*IPO8*	Hs00183533_m1	4331182
*HPRT1*	Hs99999909_m1	4333768
*NANOG*	Hs04260366_g1	4331182
*SOX2*	Hs04234836_s1	4351372
*POU5F1/OCT4*	Hs04260367_gH	4331182
*KLF4*	Hs00358836_m1	4331182
*AGR2*	Hs00356521_m1	4331182
*NOTCH1*	Hs01062014_m1	4331182

**Table 2:** miScript primer assays.

miRNA symbol	Assay ID	Catalogue no.
*SNORA73A*	Hs_SNORA73A_11	MS00014021
*SNORD72*	Hs_SNORD72_11	MS00033719
*SNORD61*	Hs_SNORD61_11	MS00033705
*SNORD95*	Hs_SNORD95_11	MS00033726
*miR-145*	Hs_miR-145_1	MS00003528
*miR-335*	Hs_miR-335_1	MS00003976
*miR-150*	Hs_miR-150_1	MS00003577
*miR-34a*	Hs_miR-34a_1	MS00003318
*miR-128*	Hs_miR-128_1	MS00008582
*miR-27a*	Hs_miR-27a_1	MS00003241

## Data Availability

The datasets used and analyzed during the current study are available from the corresponding author on reasonable request.

## Supplementary Materials

Supplementary materials Supplementary file 1: Table S1: list of primers for methylation analyses of NANOG promoter. Supplementary file 1: Table S2: correlation between expression of mRNAs and miRNAs in RNAlater-stored tumor tissue (*n* = 82). Abbreviations: ^∗^correlation is significant at the *p* ≤ 0.05 level (2-tailed); ^∗∗^correlation is significant at the *p* ≤ 0.01 level (2-tailed); ^∗∗∗^correlation is significant at the *p* ≤ 0.001 level (2-tailed). Supplementary file 1: Figure S1: negative NANOG staining in morphologically normal mucosa. Immunohistochemistry, orig. magnification, 20x. Supplementary file 1: Figure S2: promoter region of NANOG-201 transcript (ENST00000229307.9; >12 dna:chromosome:GRCh38:12:7787400:7791801:1). All analyzed CpGs are colored light blue and numbered. CpGs 22 (A->G) and 29 (T->G) are colored green, since we noticed the difference compared to reference sequence. Exon 1 is colored blue with signed TATA box and red thymine, transcription start site. Supplementary file 1: Figure S3: the color scaled ratio of samples with differences in methylation status for each CpG comparing tumor and tumor-adjacent normal tissue. Different shades represent the percentage of methylation, with red shades CpGs with no methylation and with green shades completely methylated and with shades between red and green all other percentages of methylation difference. Supplementary file 1: Figure S4: NANOG copy number variation in tumor (T) and tumoradjacent normal tissue (N) for all the RNAlater-stored samples (*n* = 87).
