# Supplementary material for: Expression of NANOG and Its Regulation in Oral Squamous Cell Carcinoma
Source: Biomed Res Int. 2020 Jul 17;2020:8573793. doi: 10.1155/2020/8573793 (PMC7383335; doi:10.1155/2020/8573793)
Supplement: Supplementary materials — Supplementary file 1: Table S1: list of primers for methylation analyses of NANOG promoter. Supplementary file 1: Table S2: correlation between expression of mRNAs and miRNAs in RNAlater-stored tumor tissue (n = 82). Abbreviations: ∗correlation is significant at the p ≤ 0.05 level (2-tailed); ∗∗correlation is significant at the p ≤ 0.01 level (2-tailed); ∗∗∗correlation is significant at the p ≤ 0.001 level (2-tailed). Supplementary file 1: Figure S1: negative NANOG staining in morphologically normal mucosa. Immunohistochemistry, orig. magnification, 20x. Supplementary file 1: Figure S2: promoter region of NANOG-201 transcript (ENST00000229307.9; >12 dna:chromosome:GRCh38:12:7787400:7791801:1). All analyzed CpGs are colored light blue and numbered. CpGs 22 (A->G) and 29 (T->G) are colored green, since we noticed the difference compared to reference sequence. Exon 1 is colored blue with signed TATA box and red thymine, transcription start site. Supplementary file 1: Figure S3: the color scaled ratio of samples with differences in methylation status for each CpG comparing tumor and tumor-adjacent normal tissue. Different shades represent the percentage of methylation, with red shades CpGs with no methylation and with green shades completely methylated and with shades between red and green all other percentages of methylation difference. Supplementary file 1: Figure S4: NANOG copy number variation in tumor (T) and tumoradjacent normal tissue (N) for all the RNAlater-stored samples (n = 87). [file 8573793.f1.pdf]

Fragment	Forward primer sequence	Reverse primer sequence	Primer for sequencing	Methylation sites in promotor
1	5'-GTATAGATAGGGTTTTATTATGTTGG-3'	5'-AAAAAAAACTTACCCTCCTATCTCT-3'	Forward	1-6
2	5'- GGTTTTTATTTTAGATATAATGGGATAGG-3'		Reverse	7-10
3	5'-AGAGATAGGAGGGTAAGTTTTTTTT-3'	5'-ATAATTTTTAAAAACAAAACTCTTTAACTC-3'	Reverse	11-15
4	5'-GAATAAAAGTTAGTTTGTGTGGGAGT-3'	5'-CCATTTTCCTCTTTCATTCACTCT-3'	Forward	16-18
			Reverse	19-21
5	5'-AGAGTGAATGAAAGAGGAAAATGG-3'	5'-TCCAACTTTTAAATCAAAAATATAATTCAAC-3'	Forward	22
			Reverse	23-35
6	5'-AGAGTAGTTGGGATTATAGATATTTA-3'	5'-TAACATAAAACAACCAACTCAATCC-3'	Reverse	36-39
7	5'-GTTGAATTATATTTTTGATTTAAAAGTTGGA-3'	5'-CATAAAACAACCAACTCAATCC-3'	Forward	40
8	5'-GGGTGGGTTTAAGGTGATAGAG-3'	5'-TCAAACCCACAAATCACAAACA-3'	Forward	41

Table S1. List of primers for methylation analyses of NANOG promoter.

		T status of samples	N status of samples	T and N status of samples	*NANOG*	*SOX2*	*OCT4*	*KLF4*	*AGR2*	*NOTCH1*	*miR - 145*	*miR - 335*	*miR - 150*	*miR - 34a*	*miR - 128*	*miR - 27a*
T status of samples	Correlation Coefficient	1	0.240*	0.932***	-0.06	-0.115	0.076	0.004	0.101	0.06	0.065	0.236*	0.219*	0.051	0.142	0.141
	Sig0. (2-tailed)	0.	0.03	0	0.591	0.302	0.496	0.971	0.365	0.589	0.563	0.033	0.048	0.648	0.204	0.205
	N	82	82	82	82	82	82	82	82	82	82	82	82	82	82	82
N status of samples	Correlation Coefficient	0.240*	1	0.531***	-0.013	-0.142	0.039	-0.045	-0.027	-0.043	0.124	-0.04	-0.177	0	-0.034	0.041
	Sig0. (2-tailed)	0.03	.	0	0.907	0.202	0.727	0.691	0.809	0.698	0.268	0.72	0.111	1	0.764	0.713
	N	82	82	82	82	82	82	82	82	82	82	82	82	82	82	82
T and N status of samples	Correlation Coefficient	0.932**	0.531**	1	-0.081	-0.15	0.079	-0.026	0.094	0.036	0.084	0.177	0.089	0.03	0.081	0.135
	Sig0. (2-tailed)	0	0	.	0.467	0.179	0.483	0.816	0.4	0.751	0.453	0.112	0.428	0.789	0.468	0.227
	N	82	82	82	82	82	82	82	82	82	82	82	82	82	82	82
NANOG	Correlation Coefficient	-0.06	-0.013	-0.081	1	0.292**	0.395***	0.570***	0.219*	0.515***	0.132	0.149	0.187	0.261*	0.069	0.068
	Sig0. (2-tailed)	0.591	0.907	0.467	.	0.008	0	0	0.048	0	0.237	0.182	0.093	0.018	0.538	0.543
	N	82	82	82	82	82	82	82	82	82	82	82	82	82	82	82
SOX2	Correlation Coefficient	-0.115	-0.142	-0.15	0.292**	1	0.039	0.322**	0.486***	0.12	0.1	0.036	-0.177	0.016	-0.068	-0.202
	Sig0. (2-tailed)	0.302	0.202	0.179	0.008	.	0.727	0.003	0	0.284	0.373	0.745	0.112	0.885	0.546	0.069
	N	82	82	82	82	82	82	82	82	82	82	82	82	82	82	82
OCT4	Correlation Coefficient	0.076	0.039	0.079	0.395**	0.039	1	0.318**	0.206	0.442***	0.12	0.118	0.078	0.247*	0.098	0.265*
	Sig0. (2-tailed)	0.496	0.727	0.483	0	0.727	.	0	0.063	0	0.285	0.293	0.484	0.025	0.381	0.016
	N	82	82	82	82	82	82	82	82	82	82	82	82	82	82	82
KLF4	Correlation Coefficient	0.004	-0.045	-0.026	0.570**	0.322**	0.318**	1	0.269*	0.482***	0.015	-0.055	-0.034	0.09	-0.068	0.119
	Sig0. (2-tailed)	0.971	0.691	0.816	0	0.003	0.004	.	0.015	0	0.892	0.625	0.759	0.419	0.546	0.288
	N	82	82	82	82	82	82	82	82	82	82	82	82	82	82	82
AGR2	Correlation Coefficient	0.101	-0.027	0.094	0.219*	0.486**	0.206	0.269*	1	0.103	0.134	0.102	-0.043	0.164	0.052	0.1
	Sig0. (2-tailed)	0.365	0.809	0.4	0.048	0	0.063	0.015	.	0.357	0.231	0.362	0.703	0.142	0.644	0.374
	N	82	82	82	82	82	82	82	82	82	82	82	82	82	82	82
NOTCH1	Correlation Coefficient	0.06	-0.043	0.036	0.515**	0.12	0.442**	0.482**	0.103	1	0.233*	0.254*	0.428***	0.290**	0.13	0.250*
	Sig0. (2-tailed)	0.589	0.698	0.751	0	0.284	0	0	0.357	.	0.035	0.021	0	0.008	0.244	0.024
	N	82	82	82	82	82	82	82	82	82	82	82	82	82	82	82
miR - 145	Correlation Coefficient	0.065	0.124	0.084	0.132	0.1	0.12	0.015	0.134	0.233*	1	0.691***	0.253*	0.796***	0.592***	0.646***
	Sig0. (2-tailed)	0.563	0.268	0.453	0.237	0.373	0.285	0.892	0.231	0.035	.	0	0.022	0	0	0
	N	82	82	82	82	82	82	82	82	82	82	82	82	82	82	82
miR - 335	Correlation Coefficient	0.236*	-0.04	0.177	0.149	0.036	0.118	-0.055	0.102	0.254*	0.691**	1	0.415***	0.641***	0.552***	0.548***
	Sig0. (2-tailed)	0.033	0.72	0.112	0.182	0.745	0.293	0.625	0.362	0.021	0	.	0	0	0	0
	N	82	82	82	82	82	82	82	82	82	82	82	82	82	82	82
miR - 150	Correlation Coefficient	0.219*	-0.177	0.089	0.187	-0.177	0.078	-0.034	-0.043	0.428**	0.253*	0.415**	1	0.376**	0.337**	0.265*
	Sig0. (2-tailed)	0.048	0.111	0.428	0.093	0.112	0.484	0.759	0.703	0	0.022	0	.	0.001	0.002	0.016
	N	82	82	82	82	82	82	82	82	82	82	82	82	82	82	82
miR - 34a	Correlation Coefficient	0.051	0	0.03	0.261*	0.016	0.247*	0.09	0.164	0.290**	0.796**	0.641**	0.376**	1	0.606***	0.765***
	Sig0. (2-tailed)	0.648	1	0.789	0.018	0.885	0.025	0.419	0.142	0.008	0	0	0.001	.	0	0
	N	82	82	82	82	82	82	82	82	82	82	82	82	82	82	82
miR - 128	Correlation Coefficient	0.142	-0.034	0.081	0.069	-0.068	0.098	-0.068	0.052	0.13	0.592**	0.552**	0.337**	0.606**	1	0.567***
	Sig0. (2-tailed)	0.204	0.764	0.468	0.538	0.546	0.381	0.546	0.644	0.244	0	0	0.002	0	.	0
	N	82	82	82	82	82	82	82	82	82	82	82	82	82	82	82
miR - 27a	Correlation Coefficient	0.141	0.041	0.135	0.068	-0.202	0.265*	0.119	0.1	0.250*	0.646**	0.548**	0.265*	0.765**	0.567**	1
	Sig0. (2-tailed)	0.205	0.713	0.227	0.543	0.069	0.016	0.288	0.374	0.024	0	0	0.016	0	0	.
	N	82	82	82	82	82	82	82	82	82	82	82	82	82	82	82

Table S2. Correlation between expression of mRNAs and miRNAs in RNAlater stored tumor tissue (n=82). Abbreviations: * Correlation is significant at the p≤0.05 level (2-tailed); ** Correlation is significant at the p≤0.01 level (2-tailed); *** Correlation is significant at the p≤0.001 level (2-tailed).

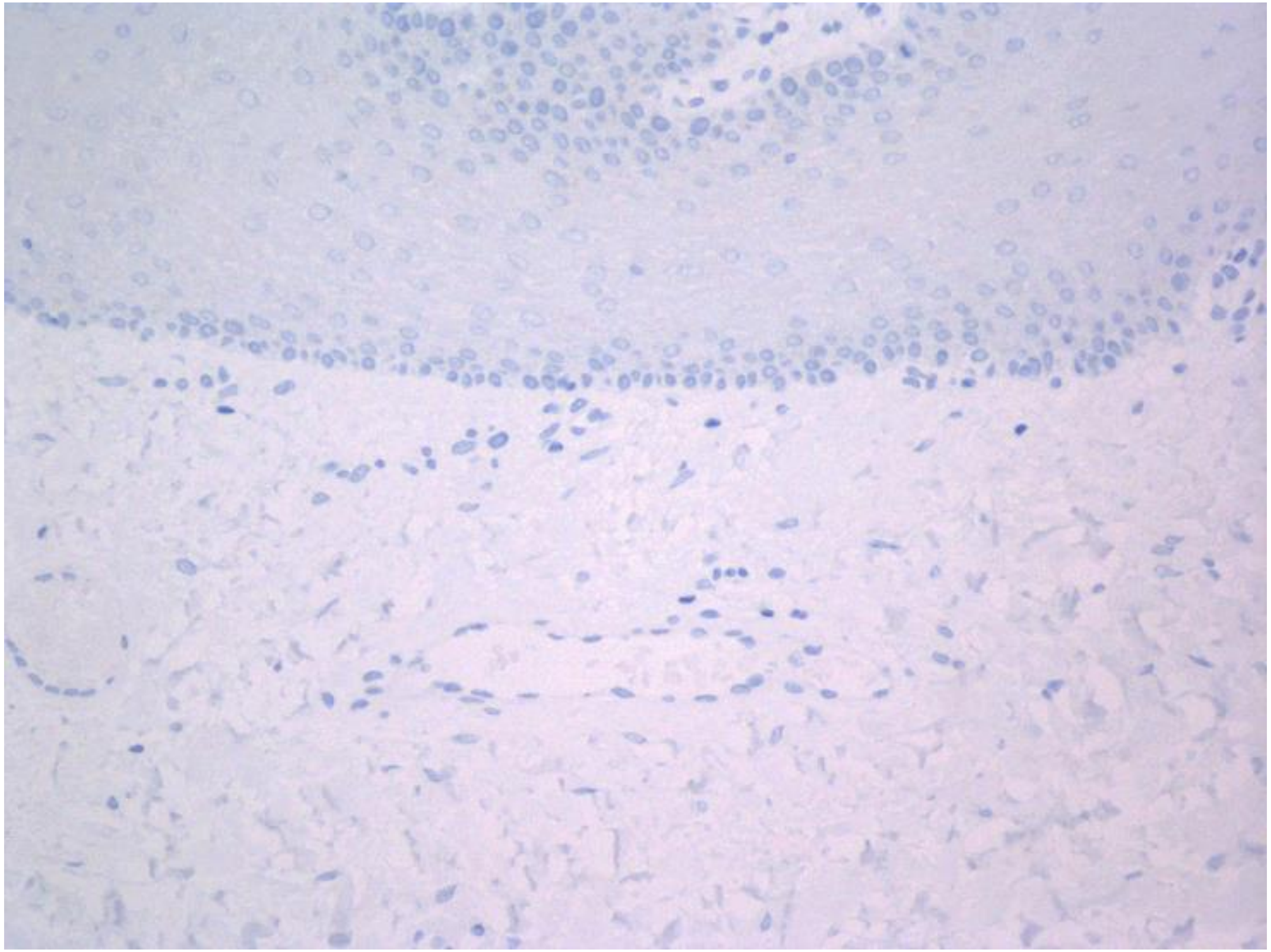

Figure S1. Negative NANOG staining in morphologically normal mucosa. Immunohistochemistry, orig. magnification, 20x.

```
CCCAGTTAATTTTTGTATTTTTAGTACAGACAGGGTTTCACCATGTTGGTCAGGCTGGTCTTGAACTCCTGAT
CTCAGATGATGCACCTGGCTCGGCCTCGCAAAGTGCTGGGATTACAGGTGTGAGCCACCGTGCCCAGCCGTT
                   1    2                             3      4
AGCTCATTTTAACACATCCTTAGTCCAGCCTGTTCCAAAAAATCTAAAGTCAGATAGCTTCCTAAACCTCAA
CTTTATTCCAATTGCTTTCCTTGGCGAAGAATGTAGTAAGTCGGCCTTCCAGCCACCAGCCCCTTCCCTTTGG
                        5                6
TCTTTCACTCCGGAGGCTCTTACCCTAGACACAATGGGACAGGGAGCGGGGGATGGGGGAATTCAGCTCAG
          7                                   8
GCTTTTATGCAAAGACCCCCTTCTGCAAAGAACAAAGCTTCTGGTACCTGCCCTTTGGAGAGCTGCGGGCAA
                                                                9
GCTCAGCCTCGGTGAGTCTTGGTGGCCTTGACAGCCCCCACTTAACAAACTGTGCTGATTAAGAGAGACAG
         10
GAGGGCAAGTTTTTCCCTTCTTTTAAAGAAATCATCCTATTTCCTACGAGACATAGACTATCTGCCTGAAGC
                                              11
ATGATGTACTAGCCCCACTCACCGGCTCCCTGATGCCCCTATGCTTAATCTTCTCCGGAATGGTAGTCTGAG
                      12                               13
AAGAAAAAAGATTACGCCCAATTTCATTTCCTTGTTTCACATCAAGCAATACTTTTCGAGTCTTTGCATTGTG
              14                                        15
AACAAAAGTCAGCTTGTGTGGGAGCAAAGCCAGCTGCTCTGGGTGCAGACCCAGGAGCAGAGTGCAGAGG
AGAATGAGTCAAAGAGTTTTGTCTTCAAAAATTACATAATCGGGATTTGCTAAGAGTTTACTTTTCGGTATG
                                        16                       17
GAAGACTGGAAAAGAGAAAGAAATCTTAGGTTTCTTGAATGTTGGGTTTGGGAATAGGAAGGAAAATCGAA
                                                                   18
AACTGTAGACTTTGTCCATAAATGTTAGTGCTGGAACCCCACTCTAAAAACTTTGTTCCTTTGGAAAACACC
TCCCTTCCCCCAGAAACACACACACCCACACGAGATGGGCACGGAGTAGTCTTGAAAGACATGACAAATCA
                              19         20
CCAGACCTGGGAAGAAGCTAAAGAGCCAGAGGGAAAAAGCCAGAAGTCGACTACCTGGGAGGAGGGATA
                                               21
GACAAGAAACCAAACTAAAGGAAACTAAGGTAGGTGCTGAAAACAAGTACCATTTTCAACATTAACTGATG
CCTTGGCTTCATGCTATAATGCCATGTTGTGTTTCACTATAACCTCAGAGTGAATGAAAGAGGAAAATGGAG
CTAGTTGAAATTTCTGCCTAAACTAGCCAGATTTTGAGACACTAAGTTATCTCAAATCAAGAAATCACCCTA
ATGAGAATTTCAATAACCTCAGGAATTTAAGGTGCATGCATCCCCCGCCCCCCCCTTTTTTTTTGAGACGTA
                                              22                     23
GTCCCGCTCTGTTGCCCAGGCTGGAGTACAGTGGCGCGATATCGGCTCACCACAACCTCTGCCTCCCAGGTT
    24                            25 26    27
CAAGGGATTCTCCCGCCTCAGCTTCCAGAGTAGCTGGGACTACAGACACCCACCACCACGCGTGGCTAATTT
             28                                             29 30
TTGTATTTTTAGTAGAGAGGGGGTTTCGCCATGTTGGCCAGGCTGGTTTCAAACTCCTGACTTCAGGTGATC
                          31
CGCCTGCCACGGCCTCCCAATTTACTGGGATTACAGGGGTGGGCCACCGCGCCCGGCCTTTTTCTTAATTTTT
32       33                                     34 35  36
AAAAATATTAAAGTTTTATCCCATTCCTGTTGAACCATATTCCTGATTTAAAAGTTGGAAACGTGGTGAACC
                                                              37
TAGAAGTATTTGTTGCTGGGTTTGTCTTCAGGTTCTGTTGCTCGGTTTTCTAGTTCCCCACCTAGTCTGGGTTA
                                          38
CTCTGCAGCTACTTTTGCATTACAATGGCCTTGGTGAGACTGGTAGACGGGATTAACTGAGAATTCACAAGG
                                               39
GTGGGTCAGTAGGGGGTGTGCCCGCCAGGAGGGGTGGGTCTAAGGTGATAGAGCCTTCATTATAAATCTAG
                      40                                 Exon 1
AGACTCCAGGATTTTAACGTTCTGCTGGACTGAGCTGGTTGCCTCATGTTATTATGCAGGCAACTCACTTTAT
                  41
CCCAATTTCTTGATACTTTTCCTTCTGGAGGTCCTATTTCTCTAACATCTTCCAGAAAAGTCTTAAAGCTGCCT
TAACCTTTTTTCCAGTCCACCTCTTAAATTTTTTCCTCCTCTTCCTCTATACTAACATGAGTGTGGATCCAGCT
TGTCCCCAAAGCTTGCCTTGCTTTGAAGCATCCGACTGTAAAGAATCTTCACCTATGCCTGTGATTTGTGGGC
CTGAAGAAAACTATCCATCCTTGCAAATGTCTTCTGCTGAGATGCCTCACACGGAGACTGGTAAGAAAGAA
                                                             Intron 1
ATTTATCCTTGAAAGGCCAAGTTCCTTAAGGGAAAAGAGAGAAGGAGAGAGGGTTAAGGGATCATTT …
```

Figure S2. Promoter region of NANOG-201 transcript (ENST00000229307.9; >12 dna:chromosome:GRCh38:12:7787400:7791801:1). All analyzed CpGs are colored light blue and numbered. CpGs 22 (A->G) and 29 (T->G) are colored green, since we noticed the difference compared to reference sequence. Exon 1 is colored blue with signed TATA box and red thymine, transcription start site.

1	2	3	4	5	6	7	8	9	10
**56**	**33**	**78**	**33**	**10**	**40**	**40**	**70**	**70**	**70**

11	12	13	14	15	16	17	18	19	20
**80**	**80**	**80**	**50**	**30**	**75**	**75**	**63**	**60**	**60**

21	22	23	24	25	26	27	28	29	30
**40**	**80**	**0**	**0**	**0**	**0**	**0**	**0**	**10**	**30**

31	32	33	34	35	36	37	38	39	40
**10**	**40**	**40**	**0**	**0**	**0**	**70**	**30**	**80**	**40**

41	Number of the methylation site
**63**	Ratio of samples with the difference in methylation status comparing tumor-adjacent normal tissue and tumor tissue [%]

Figure S3. Color scaled ratio of samples with differences in methylation status for each CpG comparing tumor and tumor-adjacent normal tissue. Different shades represent the percentage of methylation, with red shades CpGs with no methylation and with green shades completely methylated and with shades between red and green all other percentages of methylation difference.

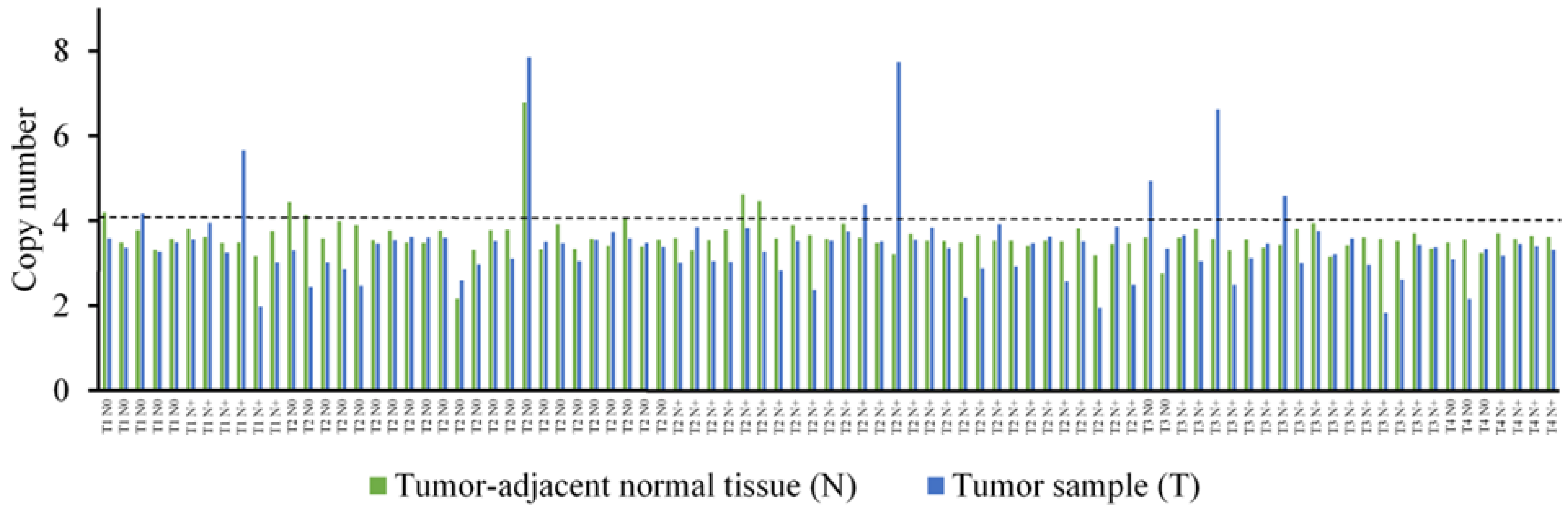

Figure S4. NANOG copy number variation in tumor (T) and tumor-adjacent normal tissue (N) for all the RNAlater stored samples (n=87).
